# Characterization of hepatitis E virus infection in tree shrew *(Tupaia belangeri chinensis)*

**DOI:** 10.1186/s12879-016-1418-1

**Published:** 2016-02-16

**Authors:** Wenhai Yu, Chenchen Yang, Yanhong Bi, Feiyan Long, Yunlong Li, Jue Wang, Fen Huang

**Affiliations:** Medical Faculty, Kunming University of Science and Technology, Kunming, China; Institute of Medical Biology, Chinese Academy of Medical Sciences and Peking Union Medical College, Kunming, China

## Abstract

**Background:**

Hepatitis E virus (HEV) is a major cause of hepatitis in developing countries and poses a threat to public health worldwide. Tree shrew (*Tupaia belangeri chinensis)* is a useful animal model in studies on hepatitis viruses, such as hepatitis B and C viruses. However, the use of this animal model for HEV research is yet to be developed.

**Methods:**

Tree shrews were intravenously (IV) injected with swine genotype 4 HEV or infected by contact-exposure to IV infected tree shrews. RT-nPCR was performed to detect HEV RNA in the feces, tissues, and blood. HEV capsid protein in the different tissues was detected by Western blot and estimated by quantitative RT-PCR. Anti-HEV antibodies were determined by ELISA. Liver damages were evaluated by histopathologic examination and analysis of liver-specific enzymes activities.

**Results:**

Both negative and positive strands of HEV RNA were detected in the feces of the HEV-infected or contact-exposed tree shrews 3–4 days post-inoculation. HEV RNA was detectable in the liver, spleen, kidneys, and bile. Virusemia developed in all the HEV-infected tree shrews. HEV capsid protein was expressed in the liver, spleen, and kidneys. The histological examination and analysis of liver-specific enzymes activities showed that HEV caused acute liver lesions in the tree shrews. Meanwhile, the infected tree shrews showed positive IgG and IgM antibodies.

**Conclusions:**

Tree shrews are susceptible to HEV and may be useful animal models for HEV experimental infection studies on pathogenesis or preclinical drug development.

## Background

Hepatitis E virus (HEV) is the most common cause of acute hepatitis worldwide. It infects 20 million people and claims 70,000 lives annually [1]. HEV infection is recognized as an emerging public health issue on a global scale. It is thought to be a zoonotic virus, the reservoirs of which are domestic animals. HEV can infect human and several animal species, such as pigs, deer, wild boars, rats, chickens, mongooses, and rabbits [2]. It is mainly transmitted via the fecal–oral route through water or food contamination. Blood transfusion, vertical transmission, and organ transplantation also transmit HEV [3].

Molecular and biological studies on HEV are limited because of the previous inability to propagate the virus efficiently in vitro. Despite the recent successes in adapting HEV to grow in cell culture systems, the viral titer of the inoculum remains limited [4]. Thus, an efficient animal model is an important tool to study HEV replication and the mechanisms of its pathogenesis. Although HEV infection animal models using non-human primates and pigs have been successfully established, these animals are large, costly, and difficult to handle. HEV has also been efficiently replicated in Balb/C nude mice [5], but such replication has not been achieved in C57BL/6 mice [6].

Tree shrew, also referred to as a *Tupaia belangeri*, belongs to the family Tupaiidae. Phylogenetically, tree shrews are more closely related to humans than to rodents [7]. Moreover, tree shrews are commonly used as animal infection models for virus research, especially studies on hepatitis B virus (HBV) and hepatitis C virus (HCV) [8, 9]. The course of HBV infection and the feature of HCC, which is similar to chronic HBV infection in humans, have been successfully established in tree shrew [9]. Similarly, the pathogenesis of HCV has been well characterized in tree shrews, and the manifestation of liver cirrhosis and hepatocellular carcinoma has been confirmed [8]. However, the HEV infection of tree shrew has not been reported yet. In the present study, tree shrews were inoculated with swine HEV to establish a novel infection animal model for HEV research.

## Methods

### Animals

The 15 tree shrews (male, four months old, 120–150 g) used in this experiment were obtained from a population of wild tree shrews (*T. belangeri chinensis*) from the Department of Laboratory Animals, the Institute of Medical Biology, the Chinese Academy of Medical Sciences, and the Peking Union Medical College. The Animal Care and Use Committee of the Kunming University of Science and Technology approved the study protocol and provided the guidelines for this study (2014JC004). Prior to their inoculation with HEV, all the tree shrews tested negative for HEV IgG and IgM antibodies and HEV antigens in both their sera and feces.

### Virus and inoculation

Swine HEV (Genotype 4, KM01 strain) was isolated from a village of Kunming City, Yunnan Province, China [10]. The feces were suspended in phosphate-buffered saline (PBS; pH 7.4) with 0.1 % diethyl pyrocarbonate (DEPC, to inhibit RNase in the feces) at a proportion of 10 % (w/v). The suspension was centrifuged at 12,000 × g for 10 min and then filtered through 0.22 μm microfilters before inoculation. The fecal supernatant was intravenously injected into each tree shrew at a minimum viral count of 1 × 10^5^ copies/mL, as calculated by the viral genomic titer determined by real-time quantitative PCR [11].

A total of fifteen tree shrews were randomly divided into three groups, namely, the HEV-infection group, HEV contact-exposed group, and the control group, each of which consisted of five tree shrews. The tree shrews in the HEV-infected group were intravenously injected with 0.2 ml HEV and each tree shrew housed in an individual cage. Those in the HEV-contacted group were not inoculated with HEV but were kept cohabiting with one HEV-infected tree shrew. Those in the control group were injected with PBS. The feces were collected daily and the sera weekly from each tree shrew after inoculation. Each of the tree shrews was humanely euthanized at 7, 14, 21, or 28 day post-inoculation (dpi) following the guidelines of the Care and Use of Laboratory Animals. The liver, bile, spleen, kidneys, and colon were collected for HEV antigen detection and stored at −80 °C until use. The livers were fixed in 10 % neutral buffered formalin for histopathologic examination.

### Determination of HEV antibodies by ELISA

The sera were tested for the presence of HEV-specific IgG and IgM antibodies using commercial ELISA kits (Wantai Biological Pharmacy Co., Beijing, China), which contained recombinant ORF2 peptides from the HEV genome, as well as both positive and negative controls. The sensitivity and specificity of the kits have been reported for human [12], mouse [5], and rhesus macaque [13] in previously studies. The cutoff value for the IgM assay was determined to be 0.26 (0.16 for IgG), aside from the mean OD450 values of the sera from the negative control (± standard deviation).

### Detection of negative-strand RNA of HEV by RT-nPCR

The total RNA of the feces or tissues was extracted using Trizol (Invitrogen, USA), in accordance with the manufacturer’s instructions. Reverse transcription was performed using M-MLV reverse transcriptase. Strand-specific primers were utilized to detect both positive and negative strands of HEV in the feces and tissues, as described previously [14, 15]. Exactly 5 μl of cDNA was used to separately amplify the positive or negative HEV strands with strand-specific primers by nested PCR (nPCR). False positive reactions for negative-strand RNA were excluded by amplification with cDNA without primers. The protocol was as follows: 94 °C for 2 min, followed by 94 °C for 30 s, 42 °C for 30 s, and 72 °C for 1 min, all of which were repeated for 29 cycles. The PCR products were detected via electrophoresis on agarose gel containing 0.5 μg/mL ethidium bromide.

### Quantitative determination of HEV by real-time qPCR

The copy number of HEV in the different organs was analyzed using SYBR green-based qPCR assays with HEV specific primers as described previously [11]. Briefly, the synthesized first-strand cDNA (2 μL) was added as a template. Real-time qPCR was performed under the following conditions: 95 °C for 30 s, followed by 39 cycles of 95 °C for 5 s and 60 °C for 31 s. The housekeeping gene (GAPDH) served as a loading control. Real-time qPCR was performed using an ABI PRISM 7300 real-time PCR system. A standard curve was generated from serial 10-fold dilutions of the synthetic HEV ORF2 from 10 to 1.0 × 10^8^ copies/mL. The synthetic HEV ORF2 was subjected to RT and the limited-cycle PCR step similar to that used for the RNA extracted from the stool and sera samples.

### Detection of HEV capsid protein by Western blot

The liver, spleen, kidneys, and colon were collected at 20 dpi. The distribution of the HEV antigen (capsid protein) in the different tissues was detected by Western blot. The tissues were lysed in RIPA buffer (50 mM Tris, pH 7.4, 150 mM NaCl, 5 mM EDTA, pH 8.0, 30 mM NaF, 1 mM Na_3_VO_4_, 40 mM β-glycerophosphate, 0.1 mM PMSF, protease inhibitors, 10 % glycerol and 1 % Nonidet-P40). The equivalent amount of total protein was then separated through 10 % sodium dodecyl sulfate–polyacrylamide gel electrophoresis and was electrophoretically transferred onto a nitrocellulose membrane. The non-specific-binding sites were blocked with 5 % skim milk, and the membrane was incubated with an HEV ORF2 monoclonal antibody (Millipore, MAB8003, 1:1000 dilutions) at 4 °C overnight. A goat–rabbit IgG conjugated with HRP was used as secondary antibody (Promega, America, 1:10,000 dilutions). The GAPDH protein incubated with monoclonal antibody (Proteintech, 1:10,000 dilutions) served as the loading control. The bands were exposed to X-ray films using the SuperSignal West Pico Trial Kit (Pierce, USA).

### Liver biochemistry profile in the sera

The activities of alanine transaminase (ALT), aspartate transaminase (AST), and alkaline phosphatase (ALP) in the sera were measured with an automated biochemistry analyzer (Olympus 2700, Japan).

### Histopathologic examination

The formalin-fixed livers were processed in paraffin following standard protocol. They were sectioned at a thickness of 3 μm and stained with hematoxylin and eosin. The sections from the control group served as the negative control. All the sections were examined using a Nikon Ti-E microscope (Japan).

## Results

### HEV shedding in the feces

HEV shedding in the feces is the most important manifestation of HEV infection. In this study, HEV RNA was first detected in the feces of the HEV-infected tree shrews from 3–4 dpi. The infected tree shrews manifested persistent HEV shedding until the end of the experiment (28 dpi, Table 1). As expected, all the tree shrews in the HEV contact-exposed group tested positive for HEV RNA from 3 or 5 dpi to 28 dpi.

**Table 1:** HEV RNA detection in tree shrews

Group			Strands	PBS	HEV infection	HEV contact
HEV RNA	Feces	0d	Positive	+(0/5)	+(0/5)	+(0/5)
			Negative	+(0/5)	+(0/5)	+(0/5)
		3d	Positive	+(0/5)	+(3/5)	+(0/5)
			Negative	+(0/5)	+(3/5)	+(0/5)
		5d	Positive	+(0/5)	+(5/5)	+(5/5)
			Negative	+(0/5)	+(5/5)	+(5/5)
		7d	Positive	+(0/4)	+(4/4)	+(4/4)
			Negative	+(0/4)	+(4/4)	+(4/4)
		14d	Positive	+(0/3)	+(3/3)	+(3/3)
			Negative	+(0/3)	+(2/3)	+(3/3)
		21d	Positive	+(0/2)	+(2/2)	+(2/2)
			Negative	+(0/2)	+(2/2)	+(2/2)
		28d	Positive	+(0/1)	+(1/1)	+(1/1)
			Negative	+(0/1)	+(1/1)	+(1/1)
	Liver		Positive	+(0/5)	+(5/5)	+(5/5)
			Negative	+(0/5)	+(4/5)	+(5/5)
	Bile		Positive	+(0/5)	+(5/5)	+(5/5)
			Negative	+(0/5)	+(3/5)	+(5/5)
	Spleen		Positive	+(0/5)	+(5/5)	+(5/5)
			Negative	+(0/5)	+(5/5)	+(5/5)
	Kidney		Positive	+(0/5)	+(4/5)	+(3/5)
			Negative	+(0/5)	+(4/5)	+(3/5)
	Colon		Positive	+(0/5)	+(1/5)	+(0/5)
			Negative	+(0/5)	+(0/5)	+(0/5)
	Serum		Positive	+(0/5)	+(5/5)	+(5/5)
			Negative	+(0/5)	+(5/5)	+(5/5)

An intermediate negative-strand RNA is produced by HEV when it replicates. Thus, the detection of a negative RNA strand is indicative of HEV replication [15]. In this work, the negative strand of HEV RNA, which was detectable from 3–4 dpi to 28 dpi (end of the experiment) in the HEV-infected and HEV contact-exposed groups (Table 1). However, the tree shrew inoculated with PBS tested negative for HEV RNA in the feces for the entire duration of the experiment.

### Replication of HEV in multiple organs

The liver, bile, spleen, kidney, colon, and blood of the tree shrews were collected at 7, 14, 21 and 28 dpi, and the total RNA was separately extracted. HEV RNA was detected in all the organs of the tree shrews in the HEV-infected and HEV contact-exposed groups, except for the colon, which was washed twice with PBS to exclude the contamination of feces (Table 1). This finding confirmed that, in addition to the liver, the spleen and kidney are additional HEV replication sites.

A negative-strand HEV RNA was detected in the liver and spleen samples from all the inoculated and contact-exposed tree shrews at 28 dpi. A negative-strand RNA was also detected in the kidney of the HEV-infected tree shrew at 7 dpi (4/5 in the HEV-infected group and 3/5 in the HEV contact-exposed group) (Table 1). This result indicates that HEV replicates in the kidney. Spleen is another important HEV replication site, which tested positive in the HEV-infected tree shrews, including HEV-infected and HEV contact-exposed groups. The long-term and persistent shedding of HEV may cause splenomegaly, a major clinical feature of HEV infection.

**Fig. 1:**
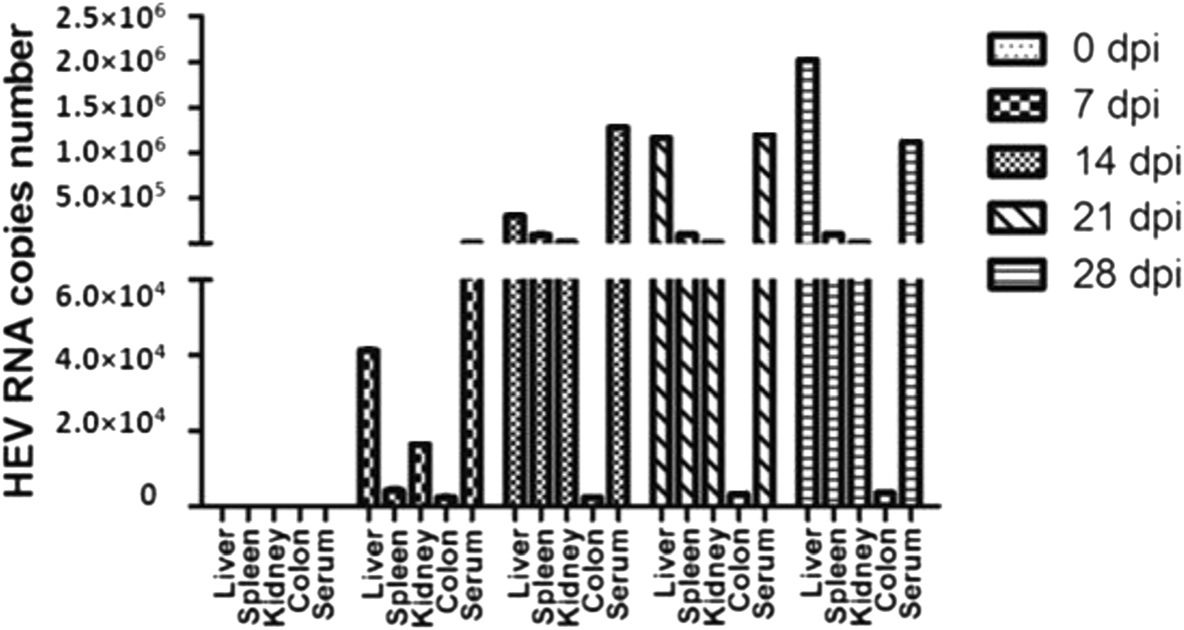
Level of HEV replication in the different organs of tree shrew intravenously injected with swine HEV was estimated by quantitative RT-PCR at 0 (1 tree shrew), 7 (1), 14 (1), 21 (1), and 28 (1) dpi

The blood samples collected at 7, 14, 21 and 28 dpi tested positive for HEV RNA (both positive and negative strands), thereby suggesting the presence of HEV in the blood circulation of the HEV-infected tree shrews for four weeks. Moreover, the level of replication in different tissues was estimated by qRT-PCR (Fig. 1). Results indicated that the liver is the main replication site of HEV and that the high amount of virus in the blood circulation is the primary source of the infection of multiple organs.

### Distribution of HEV capsid protein in different tissues

**Fig. 2:**
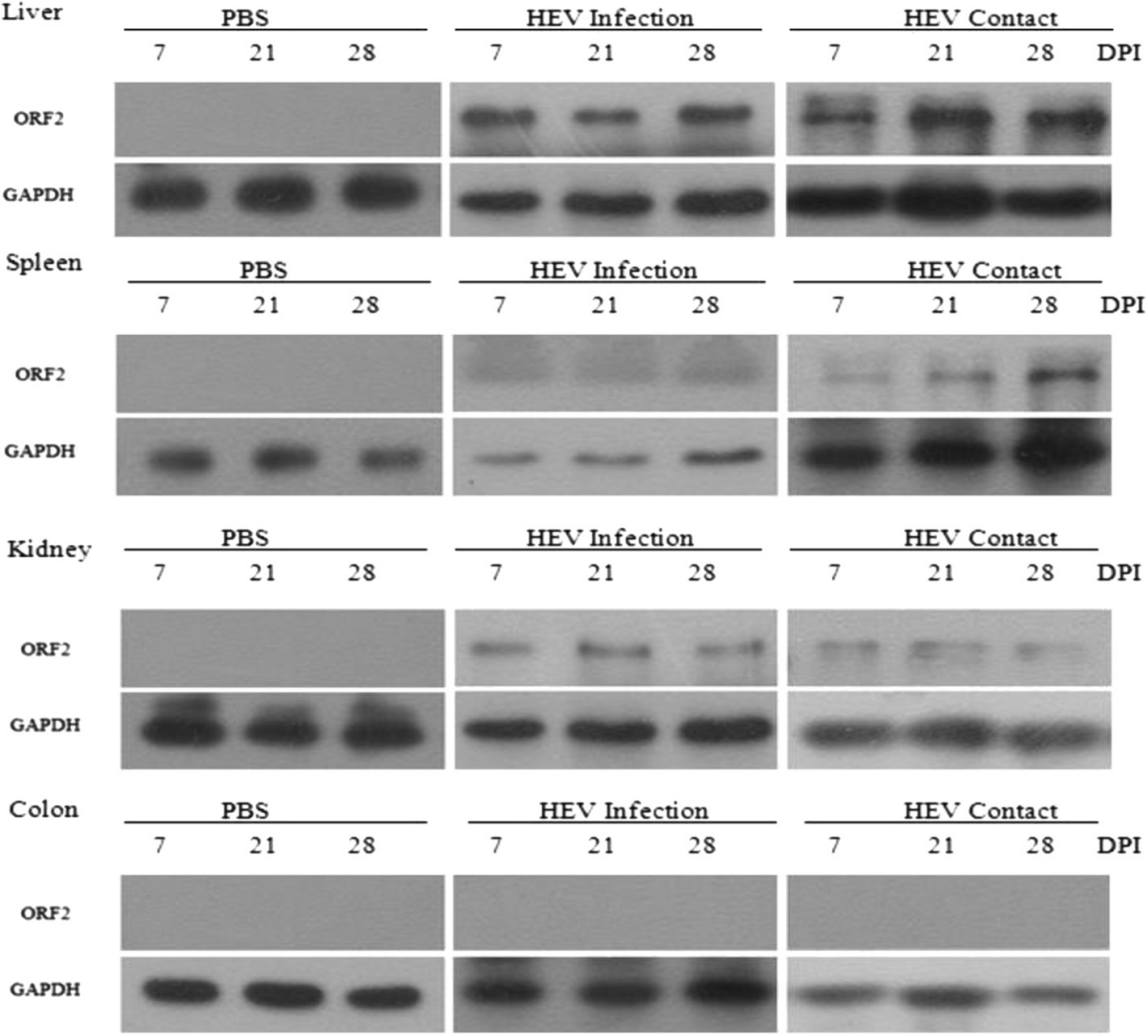
HEV capsid protein was detected by Western blot in different tissues in tree shrews inoculated with PBS (PBS), HEV (HEV Infection) or contact-exposed to HEV-infected tree shrews (HEV Contact) at 7 (three tree shrews), 21 (3), and 28 (3) dpi

Capsid protein was examined by Western blot to further analyze the antigen distribution of HEV in the different tissues at 7, 21, and 28 dpi. The HEV capsid proteins of the HEV-infected tree shrews were detected in the liver, spleen, and kidney of the specimens but not in the washed colon (Fig. 2). The highest HEV content was found in the liver, followed by the kidneys and spleen; this finding which was consistent with the results of HEV in the mRNA level.

### Humoral response induced by HEV in tree shrews

**Fig. 3:**
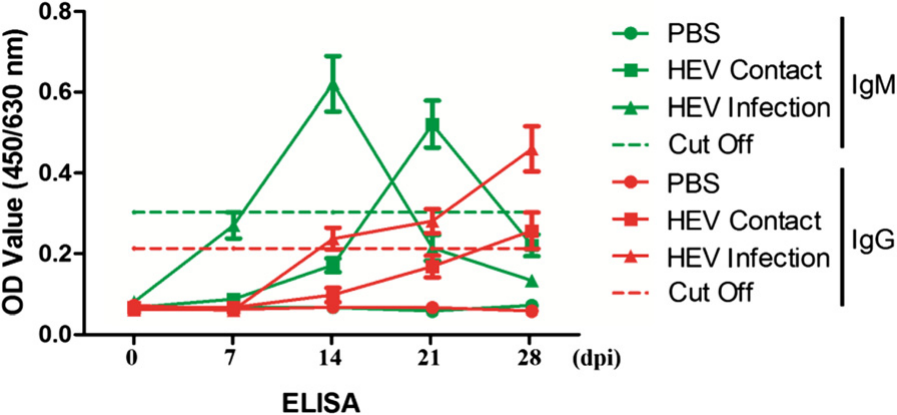
Kinetics of detection of anti-HEV IgG and IgM antibodies in tree shrew at 0 (three tree shrews), 7 (3), 14 (3), 21 (3), and 28 (3) dpi were tested by ELISA

All the HEV-infected tree shrews tested positive for HEV IgM antibodies, which indicated the occurrence of acute HEV infection. However, the IgM antibody in the contact-exposed group was elevated about one week later compared with the directly inoculated group. All the HEV-infected tree shrews tested positive for anti-HEV IgG antibodies at 28 dpi. A total of two of the three tree shrews in HEV contact-exposed group tested positive for HEV IgG at 28 dpi. All the assays were performed in triplicate, and the data were expressed as mean values (± standard deviation). The mean values of OD450 for the three groups were analyzed using the SAS system software (Fig. 3).

### Liver inflammation caused by HEV

HEV infection mainly causes the swelling and inflammation of the liver. To determine whether or not it can also cause liver injury/inflammation in tree shrews, liver-specific enzymes activities were tested. HEV infection resulted in the sharp elevation of ALT and AST, whereas no obvious change in ALP was detected.

**Fig. 4:**
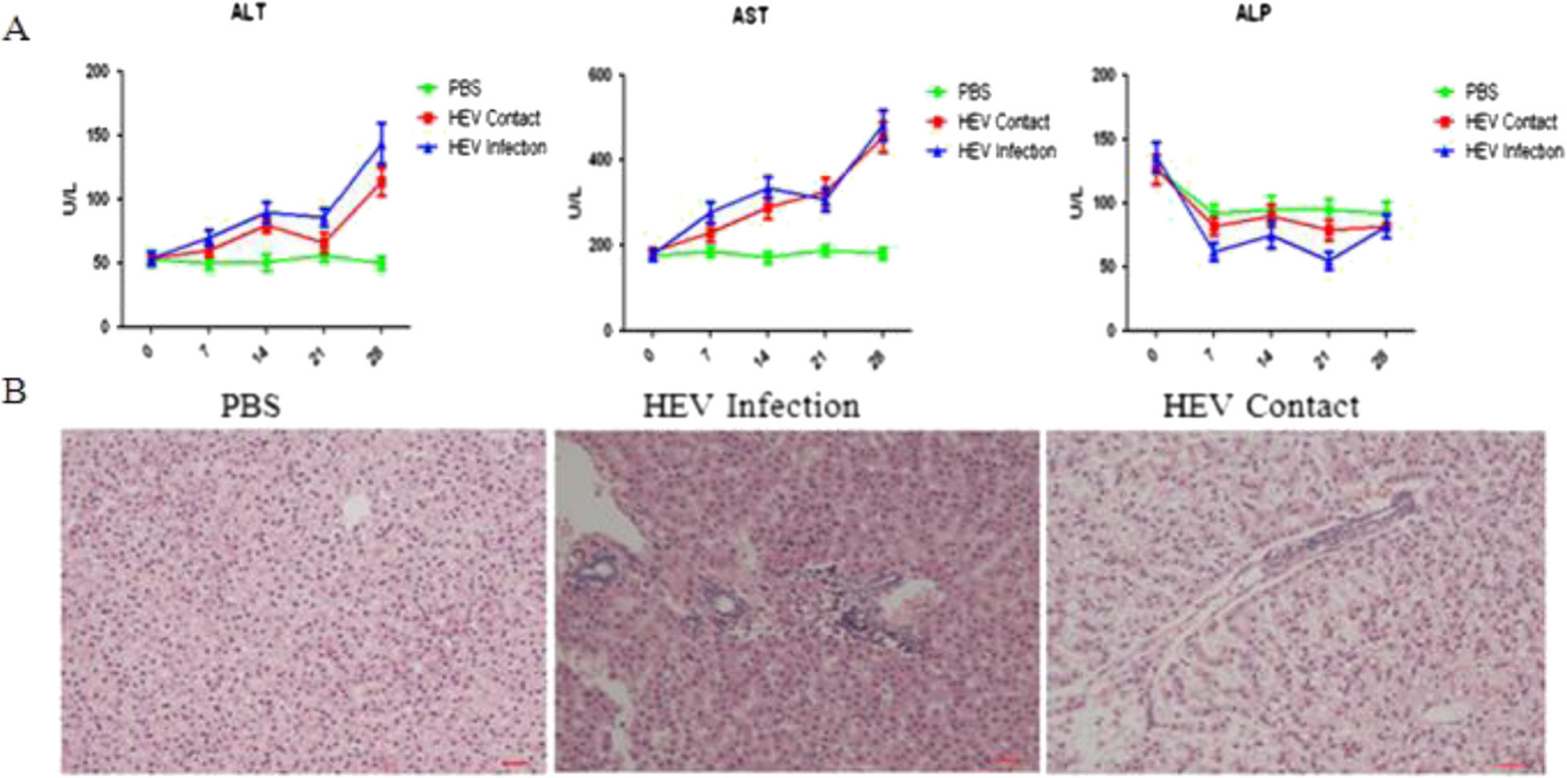
Liver inflammation caused by HEV infection. **a**: Kinetics of the level of ALT, AST, and ALP were assessed in tree shrew inoculated with PBS (PBS), HEV (HEV Infection), or contact-exposed to HEV-infected tree shrews (HEV Contact) at 0 (three tree shrews), 7 (3), 14 (3), 21 (3), and 28 (3) dpi. **b**: Liver histopathologic examination of the indicated tree shrews

The liver damage was evaluated by histopathologic examination. Increased infiltrating lymphocytes and macrophages were observed in the HEV-infected tree shrews. Hepatic inflammation, focal hepatocelluar necrosis, and liver hemorrhage were observed in all the infected tree shrews. No damage was observed in any of those in the PBS inoculation control group (Fig. 4).

## Discussion

Tree shrew is a novel animal model used in biology and virology. Although wild captured tree shrews have been spared from HEV infection [16], Asian musk shrew (*suncus murinus*), another species of shrew, is susceptible to rat HEV [17]. In the present study, the HEV-infected tree shrews manifested symptoms of HEV infection similar to HEV-infected humans. These symptoms include HEV shedding in feces (Table 1), detectable HEV antigens in the liver and other extra-hepatic tissues (Fig. 2), induced humoral response (Fig. 3), elevated liver-specific enzyme activities, and aggravated liver lesions (Fig. 4). These manifestations strongly suggest that tree shrews are susceptible to HEV. HEV RNA was first detected in the feces at 3–4 dpi; this result is consistent with that involving HEV-infected Balb/C nude mice (4 dpi) [5], swine (3 dpi) [18], or cynomolgus macaques (4–6 dpi) [19]. HEV RNA also occurred earlier in shrews than in rats inoculated with rat HEV infectious clone (14 dpi) [20]. Similarly, a negative HEV strand was detectable from 3–4 dpi to 28 dpi (i.e., end of the experiment, Table 1), which indicates that HEV RNA was shed in the feces during at least 4 weeks. HEV usually causes self-limited diseases with viral shedding in feces for 2–4 weeks [3]. HEV infection animal models showed similar syndromes. Swine HEV-infected nude mice shed virus for 3 weeks [5], and the infection of rhesus macaque lasts for 4 weeks [21]. In the present study, a large extent of HEV replication in the liver and serum was detected even at 28 dpi, suggesting that HEV replicates last for more than 28 dpi and that the HEV infection of tree shrews is successful.

HEV is replicated in multiple tissues, such as the spleen and kidneys, as confirmed by the results for Balb/C nude mice [5] and swine [22]. Similarly, HEV RNA and capsid protein are simultaneously detected in the liver, spleen, and kidneys of the HEV-infected tree shrews. This result strongly indicates that the spleen and kidney are additional extra-hepatic replication sites of HEV, and a recent study has confirmed that the virus is detectable in the urine of HEV-infected patients [21]. Although HEV antigens have been identified in the colon of HEV-infected Balb/C nude mice by indirect immunofluorescence assay [5], HEV RNA is detected in the PBS washed colon of only one tree shrew (1/3). Capsid protein is not found in any of the HEV-infected tree shrews based on the results of the Western blot. Thus, the possibility of the colon being a replication site of HEV should be further studied. HEV RNA is detected in the bile of the HEV-infected tree shrews; this result is similar to that for HEV-infected nonhuman primates or swine. Moreover, the level of ALT and AST is obviously elevated in the HEV-infected tree shrews. More interestingly, the presence of HEV antibodies (both IgM and IgG antibodies) in the tree shrews indicates that these animals can be successfully used as models for acute HEV infection studies.

Tree shrews have considerable genetic homology with both humans and primates and are therefore useful models for viral infections, including herpes simplex virus [23], rotavirus [24], and hepatitis viruses, such as HBV [9] and HCV [25]. Tree shrews are especially suitable for hepatitis studies. For example, liver cirrhosis and hepatocellular carcinoma have been observed in HCV-infected tree shrews [8]. Chronic HBV infection has also been simulated in HBV-infected tree shrews, and the results show histopathological changes similar to the clinical symptoms in HBV-infected humans [9, 26]. Furthermore, trees shrews are advantageous in anti-viral drug development and preclinical studies [27]. For instance, tree shrews have been successfully used to establish a fatty liver model [28]. Thus, tree shrews may be highly suitable animal models for HEV studies.

## Conclusions

Although further investigation on both swine genotype 3 and human genotype 1 HEV are needed, the experimental infection of tree shrews appears to be a promising approach to the investigation of HEV replication. Thus, the development of tree shrews for use as animal infection model may become a powerful tool in HEV research and in preclinical studies on drug development.

## Abbreviations

ELISA: enzyme-linked immunosorbent assay
HBV: hepatitis B virus
HCV: hepatitis C virus
HEV: hepatitis E virus
ORF: open reading fragment
RT-nPCR: reverse transcript nested polymerase chain reaction
